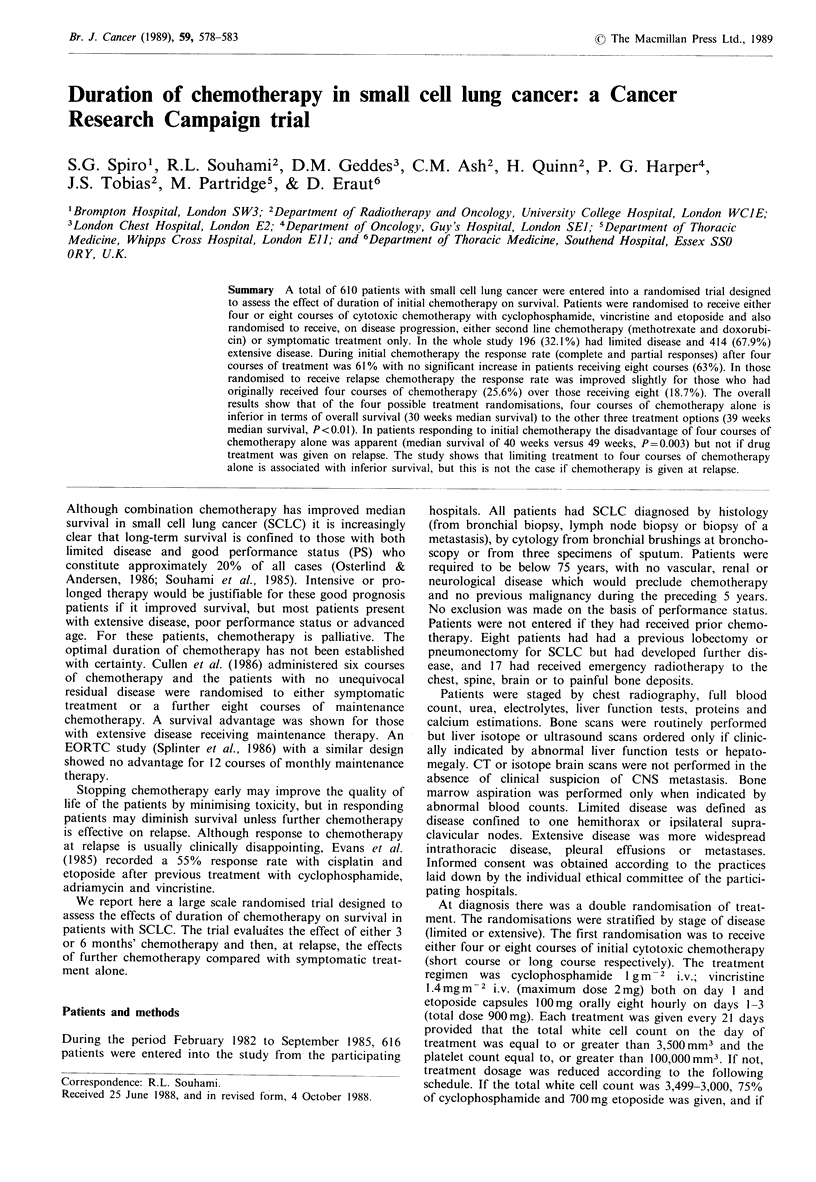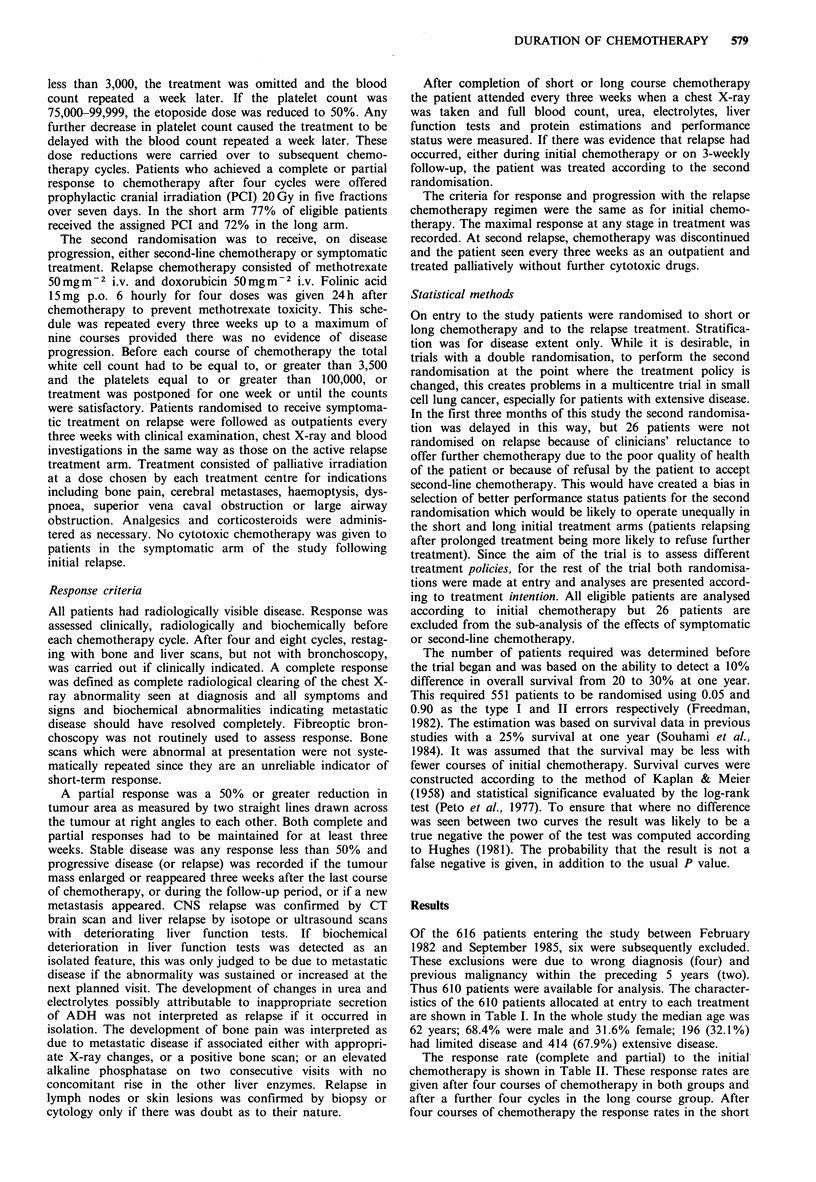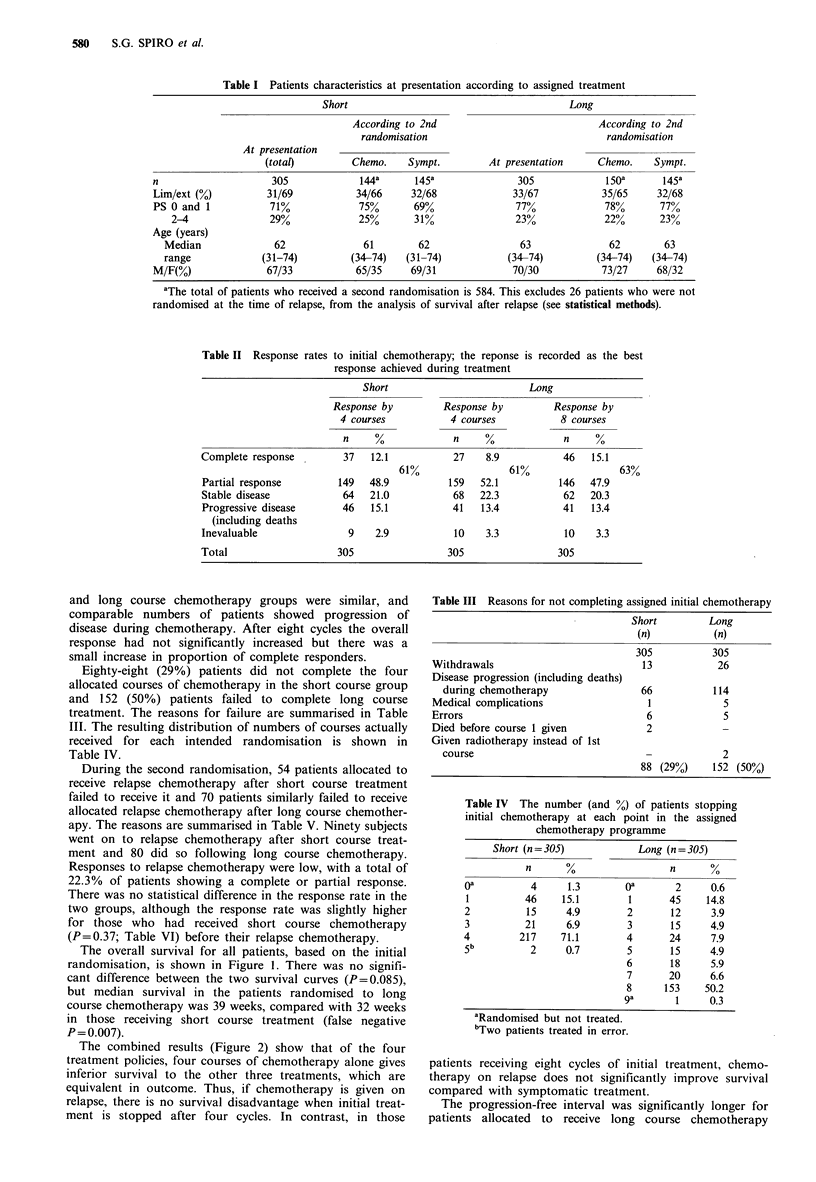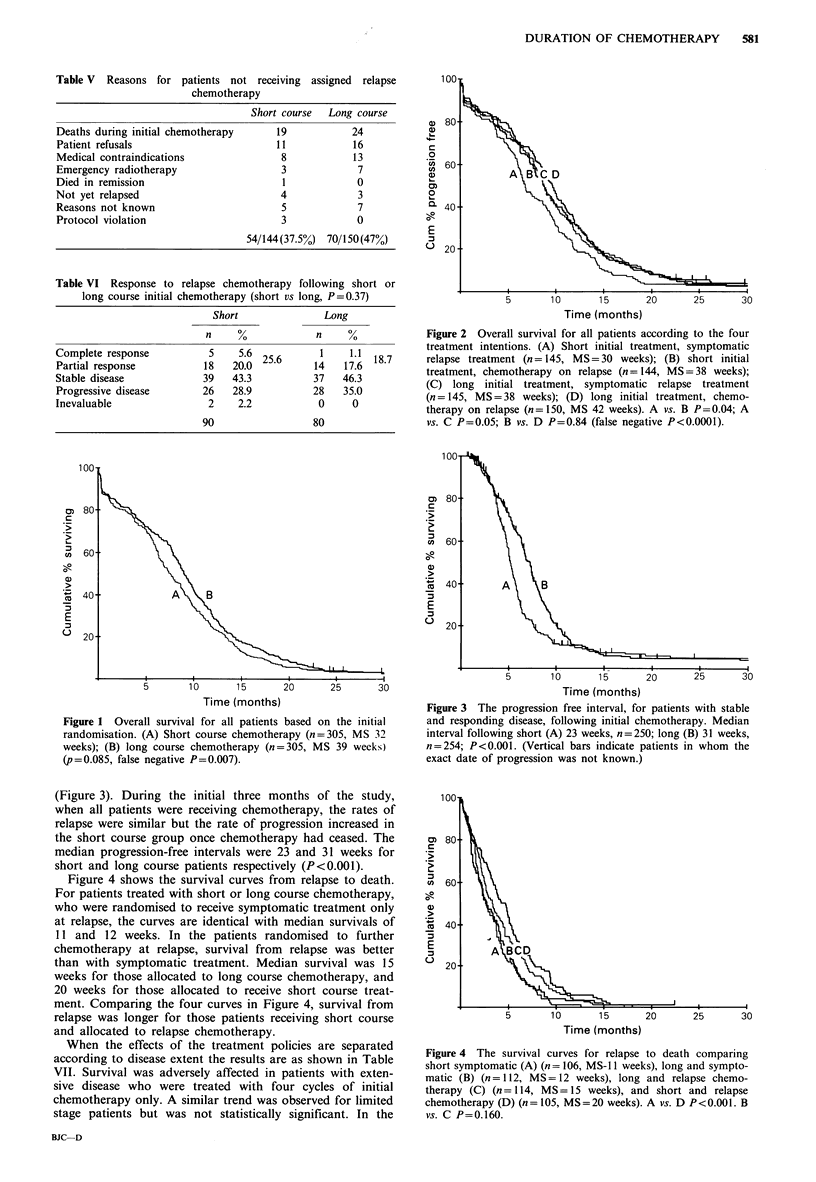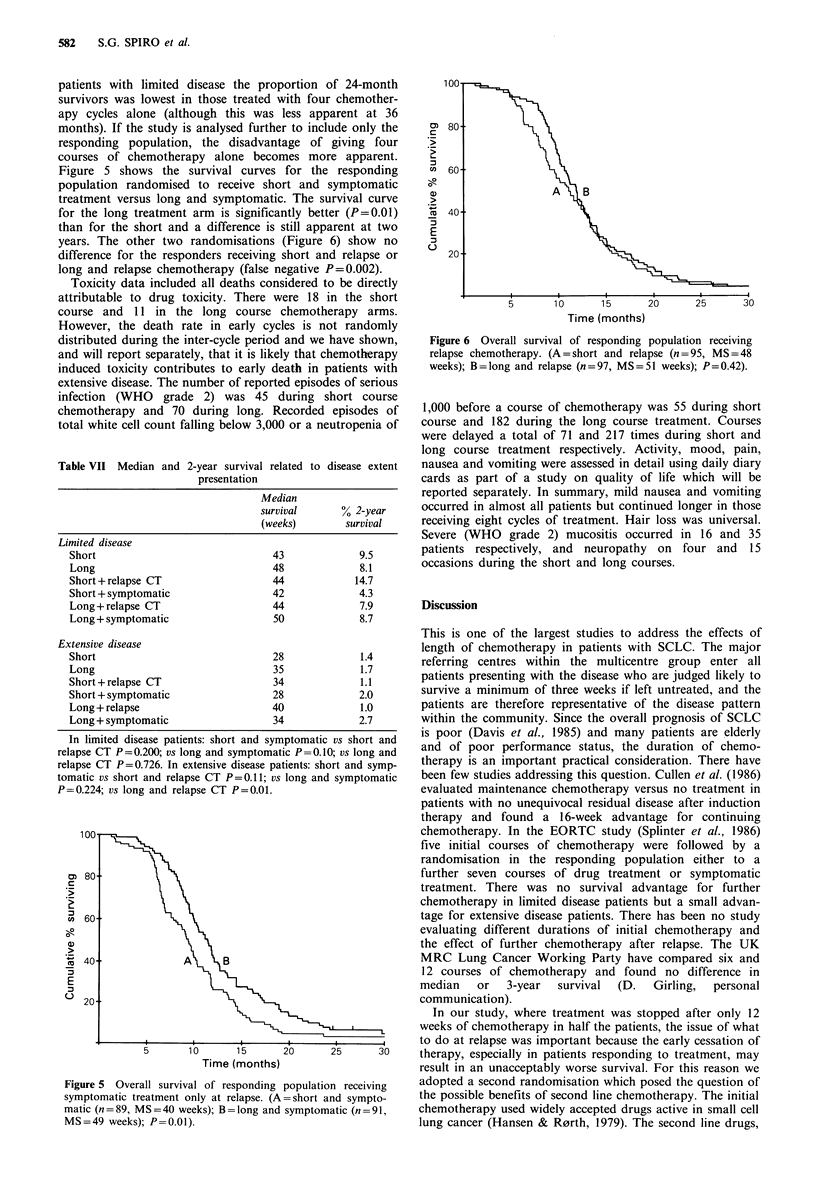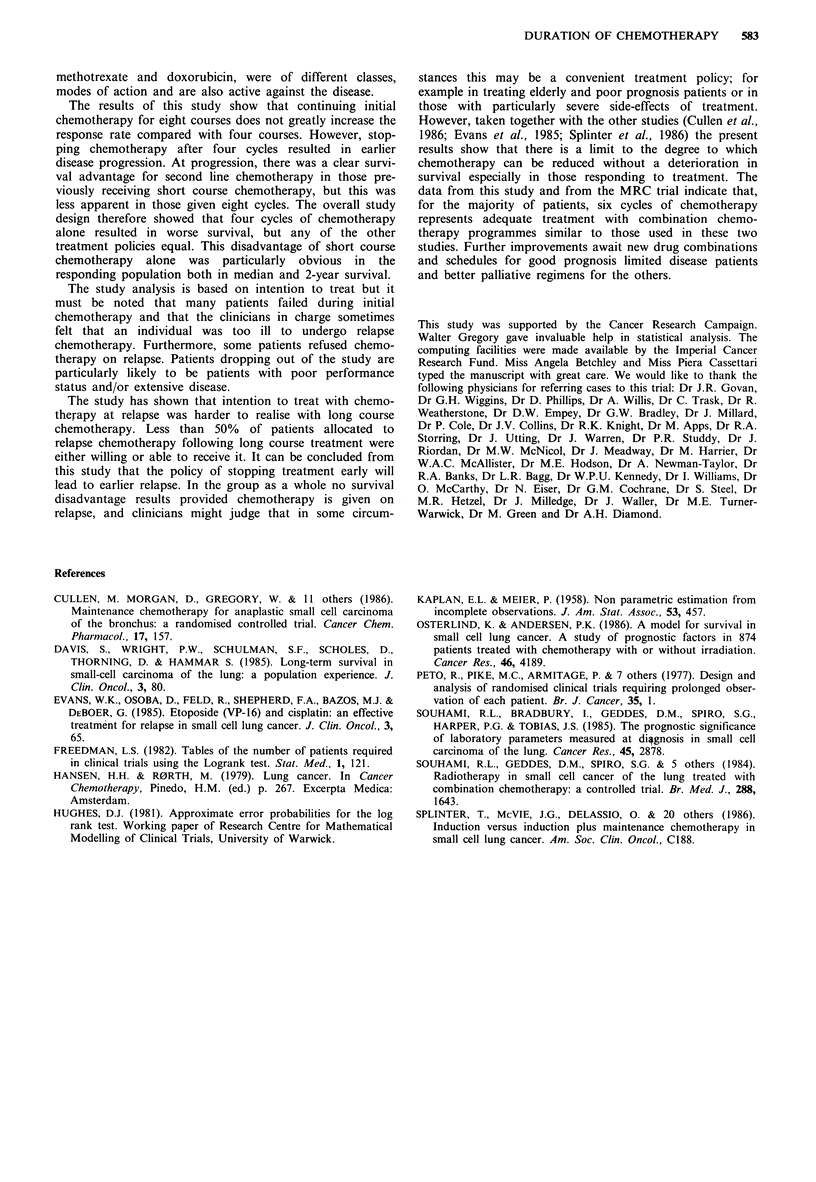# Duration of chemotherapy in small cell lung cancer: a Cancer Research Campaign trial.

**DOI:** 10.1038/bjc.1989.117

**Published:** 1989-04

**Authors:** S. G. Spiro, R. L. Souhami, D. M. Geddes, C. M. Ash, H. Quinn, P. G. Harper, J. S. Tobias, M. Partridge, D. Eraut

**Affiliations:** Brompton Hospital, London, U.K.

## Abstract

A total of 610 patients with small cell lung cancer were entered into a randomised trial designed to assess the effect of duration of initial chemotherapy on survival. Patients were randomised to receive either four or eight courses of cytotoxic chemotherapy with cyclophosphamide, vincristine and etoposide and also randomised to receive, on disease progression, either second line chemotherapy (methotrexate and doxorubicin) or symptomatic treatment only. In the whole study 196 (32.1%) had limited disease and 414 (67.9%) extensive disease. During initial chemotherapy the response rate (complete and partial responses) after four courses of treatment was 61% with no significant increase in patients receiving eight courses (63%). In those randomised to receive relapse chemotherapy the response rate was improved slightly for those who had originally received four courses of chemotherapy (25.6%) over those receiving eight (18.7%). The overall results show that of the four possible treatment randomizations, four courses of chemotherapy alone is inferior in terms of overall survival (30 weeks median survival) to the other three treatment options (39 weeks median survival, P less than 0.01). In patients responding to initial chemotherapy the disadvantage of four courses of chemotherapy alone was apparent (median survival of 40 weeks versus 49 weeks, P = 0.003) but not if drug treatment was given on relapse. The study shows that limiting treatment to four courses of chemotherapy alone is associated with inferior survival, but this is not the case if chemotherapy is given at relapse.


Br. J. Cancer (1989), 59, 578-583 © The Macmillan Press Ltd., 1989

### Duration of chemotherapy in small cell lung cancer: a Cancer Research Campaign trial

S.G. Spiro[1], R.L. Souhami[2], D.M. Geddes[3], C.M. Ash[2], H. Quinn[2], P. G. Harper[4], J.S. Tobias[2], M. Partridge[5], & D. Eraut[6]

[1]*Brompton Hospital, London SW3;* [2]*Department of Radiotherapy and Oncology, University College Hospital, London WC1E;* [3]*London Chest Hospital, London E2;* [4]*Department of Oncology, Guy's Hospital, London SE1;* [5]*Department of Thoracic Medicine, Whipps Cross Hospital, London E11; and* [6]*Department of Thoracic Medicine, Southend Hospital, Essex SS0 0RY, U.K.*

**Summary** A total of 610 patients with small cell lung cancer were entered into a randomised trial designed to assess the effect of duration of initial chemotherapy on survival. Patients were randomised to receive either four or eight courses of cytotoxic chemotherapy with cyclophosphamide, vincristine and etoposide and also randomised to receive, on disease progression, either second line chemotherapy (methotrexate and doxorubicin) or symptomatic treatment only. In the whole study 196 (32.1%) had limited disease and 414 (67.9%) extensive disease. During initial chemotherapy the response rate (complete and partial responses) after four courses of treatment was 61% with no significant increase in patients receiving eight courses (63%). In those randomised to receive relapse chemotherapy the response rate was improved slightly for those who had originally received four courses of chemotherapy (25.6%) over those receiving eight (18.7%). The overall results show that of the four possible treatment randomisations, four courses of chemotherapy alone is inferior in terms of overall survival (30 weeks median survival) to the other three treatment options (39 weeks median survival, $P<0.01$). In patients responding to initial chemotherapy the disadvantage of four courses of chemotherapy alone was apparent (median survival of 40 weeks versus 49 weeks, $P=0.003$) but not if drug treatment was given on relapse. The study shows that limiting treatment to four courses of chemotherapy alone is associated with inferior survival, but this is not the case if chemotherapy is given at relapse.

Although combination chemotherapy has improved median survival in small cell lung cancer (SCLC) it is increasingly clear that long-term survival is confined to those with both limited disease and good performance status (PS) who constitute approximately 20% of all cases (Osterlind & Andersen, 1986; Souhami *et al.*, 1985). Intensive or prolonged therapy would be justifiable for these good prognosis patients if it improved survival, but most patients present with extensive disease, poor performance status or advanced age. For these patients, chemotherapy is palliative. The optimal duration of chemotherapy has not been established with certainty. Cullen *et al.* (1986) administered six courses of chemotherapy and the patients with no unequivocal residual disease were randomised to either symptomatic treatment or a further eight courses of maintenance chemotherapy. A survival advantage was shown for those with extensive disease receiving maintenance therapy. An EORTC study (Splinter *et al.*, 1986) with a similar design showed no advantage for 12 courses of monthly maintenance therapy.

Stopping chemotherapy early may improve the quality of life of the patients by minimising toxicity, but in responding patients may diminish survival unless further chemotherapy is effective on relapse. Although response to chemotherapy at relapse is usually clinically disappointing, Evans *et al.* (1985) recorded a 55% response rate with cisplatin and etoposide after previous treatment with cyclophosphamide, adriamycin and vincristine.

We report here a large scale randomised trial designed to assess the effects of duration of chemotherapy on survival in patients with SCLC. The trial evaluates the effect of either 3 or 6 months' chemotherapy and then, at relapse, the effects of further chemotherapy compared with symptomatic treatment alone.

### Patients and methods

During the period February 1982 to September 1985, 616 patients were entered into the study from the participating hospitals. All patients had SCLC diagnosed by histology (from bronchial biopsy, lymph node biopsy or biopsy of a metastasis), by cytology from bronchial brushings at bronchoscopy or from three specimens of sputum. Patients were required to be below 75 years, with no vascular, renal or neurological disease which would preclude chemotherapy and no previous malignancy during the preceding 5 years. No exclusion was made on the basis of performance status. Patients were not entered if they had received prior chemotherapy. Eight patients had had a previous lobectomy or pneumonectomy for SCLC but had developed further disease, and 17 had received emergency radiotherapy to the chest, spine, brain or to painful bone deposits.

Patients were staged by chest radiography, full blood count, urea, electrolytes, liver function tests, proteins and calcium estimations. Bone scans were routinely performed but liver isotope or ultrasound scans ordered only if clinically indicated by abnormal liver function tests or hepatomegaly. CT or isotope brain scans were not performed in the absence of clinical suspicion of CNS metastasis. Bone marrow aspiration was performed only when indicated by abnormal blood counts. Limited disease was defined as disease confined to one hemithorax or ipsilateral supraclavicular nodes. Extensive disease was more widespread intrathoracic disease, pleural effusions or metastases. Informed consent was obtained according to the practices laid down by the individual ethical committee of the participating hospitals.

At diagnosis there was a double randomisation of treatment. The randomisations were stratified by stage of disease (limited or extensive). The first randomisation was to receive either four or eight courses of initial cytotoxic chemotherapy (short course or long course respectively). The treatment regimen was cyclophosphamide $1\,gm^{-2}$ i.v.; vincristine $1.4\,mg\,m^{-2}$ i.v. (maximum dose 2 mg) both on day 1 and etoposide capsules 100 mg orally eight hourly on days 1–3 (total dose 900 mg). Each treatment was given every 21 days provided that the total white cell count on the day of treatment was equal to or greater than $3{,}500\,mm^3$ and the platelet count equal to, or greater than $100{,}000\,mm^3$. If not, treatment dosage was reduced according to the following schedule. If the total white cell count was 3,499–3,000, 75% of cyclophosphamide and 700 mg etoposide was given, and if

Correspondence: R.L. Souhami.
Received 25 June 1988, and in revised form, 4 October 1988.

less than 3,000, the treatment was omitted and the blood count repeated a week later. If the platelet count was 75,000–99,999, the etoposide dose was reduced to 50%. Any further decrease in platelet count caused the treatment to be delayed with the blood count repeated a week later. These dose reductions were carried over to subsequent chemotherapy cycles. Patients who achieved a complete or partial response to chemotherapy after four cycles were offered prophylactic cranial irradiation (PCI) 20 Gy in five fractions over seven days. In the short arm 77% of eligible patients received the assigned PCI and 72% in the long arm.

The second randomisation was to receive, on disease progression, either second-line chemotherapy or symptomatic treatment. Relapse chemotherapy consisted of methotrexate $50 \text{mg m}^{-2}$ i.v. and doxorubicin $50 \text{mg m}^{-2}$ i.v. Folinic acid 15 mg p.o. 6 hourly for four doses was given 24 h after chemotherapy to prevent methotrexate toxicity. This schedule was repeated every three weeks up to a maximum of nine courses provided there was no evidence of disease progression. Before each course of chemotherapy the total white cell count had to be equal to, or greater than 3,500 and the platelets equal to or greater than 100,000, or treatment was postponed for one week or until the counts were satisfactory. Patients randomised to receive symptomatic treatment on relapse were followed as outpatients every three weeks with clinical examination, chest X-ray and blood investigations in the same way as those on the active relapse treatment arm. Treatment consisted of palliative irradiation at a dose chosen by each treatment centre for indications including bone pain, cerebral metastases, haemoptysis, dyspnoea, superior vena caval obstruction or large airway obstruction. Analgesics and corticosteroids were administered as necessary. No cytotoxic chemotherapy was given to patients in the symptomatic arm of the study following initial relapse.

### *Response criteria*

All patients had radiologically visible disease. Response was assessed clinically, radiologically and biochemically before each chemotherapy cycle. After four and eight cycles, restaging with bone and liver scans, but not with bronchoscopy, was carried out if clinically indicated. A complete response was defined as complete radiological clearing of the chest X-ray abnormality seen at diagnosis and all symptoms and signs and biochemical abnormalities indicating metastatic disease should have resolved completely. Fibreoptic bronchoscopy was not routinely used to assess response. Bone scans which were abnormal at presentation were not systematically repeated since they are an unreliable indicator of short-term response.

A partial response was a 50% or greater reduction in tumour area as measured by two straight lines drawn across the tumour at right angles to each other. Both complete and partial responses had to be maintained for at least three weeks. Stable disease was any response less than 50% and progressive disease (or relapse) was recorded if the tumour mass enlarged or reappeared three weeks after the last course of chemotherapy, or during the follow-up period, or if a new metastasis appeared. CNS relapse was confirmed by CT brain scan and liver relapse by isotope or ultrasound scans with deteriorating liver function tests. If biochemical deterioration in liver function tests was detected as an isolated feature, this was only judged to be due to metastatic disease if the abnormality was sustained or increased at the next planned visit. The development of changes in urea and electrolytes possibly attributable to inappropriate secretion of ADH was not interpreted as relapse if it occurred in isolation. The development of bone pain was interpreted as due to metastatic disease if associated either with appropriate X-ray changes, or a positive bone scan; or an elevated alkaline phosphatase on two consecutive visits with no concomitant rise in the other liver enzymes. Relapse in lymph nodes or skin lesions was confirmed by biopsy or cytology only if there was doubt as to their nature.

After completion of short or long course chemotherapy the patient attended every three weeks when a chest X-ray was taken and full blood count, urea, electrolytes, liver function tests and protein estimations and performance status were measured. If there was evidence that relapse had occurred, either during initial chemotherapy or on 3-weekly follow-up, the patient was treated according to the second randomisation.

The criteria for response and progression with the relapse chemotherapy regimen were the same as for initial chemotherapy. The maximal response at any stage in treatment was recorded. At second relapse, chemotherapy was discontinued and the patient seen every three weeks as an outpatient and treated palliatively without further cytotoxic drugs.

### *Statistical methods*

On entry to the study patients were randomised to short or long chemotherapy and to the relapse treatment. Stratification was for disease extent only. While it is desirable, in trials with a double randomisation, to perform the second randomisation at the point where the treatment policy is changed, this creates problems in a multicentre trial in small cell lung cancer, especially for patients with extensive disease. In the first three months of this study the second randomisation was delayed in this way, but 26 patients were not randomised on relapse because of clinicians' reluctance to offer further chemotherapy due to the poor quality of health of the patient or because of refusal by the patient to accept second-line chemotherapy. This would have created a bias in selection of better performance status patients for the second randomisation which would be likely to operate unequally in the short and long initial treatment arms (patients relapsing after prolonged treatment being more likely to refuse further treatment). Since the aim of the trial is to assess different treatment *policies,* for the rest of the trial both randomisations were made at entry and analyses are presented according to treatment *intention.* All eligible patients are analysed according to initial chemotherapy but 26 patients are excluded from the sub-analysis of the effects of symptomatic or second-line chemotherapy.

The number of patients required was determined before the trial began and was based on the ability to detect a 10% difference in overall survival from 20 to 30% at one year. This required 551 patients to be randomised using 0.05 and 0.90 as the type I and II errors respectively (Freedman, 1982). The estimation was based on survival data in previous studies with a 25% survival at one year (Souhami *et al.*, 1984). It was assumed that the survival may be less with fewer courses of initial chemotherapy. Survival curves were constructed according to the method of Kaplan & Meier (1958) and statistical significance evaluated by the log-rank test (Peto *et al.*, 1977). To ensure that where no difference was seen between two curves the result was likely to be a true negative the power of the test was computed according to Hughes (1981). The probability that the result is not a false negative is given, in addition to the usual $P$ value.

### Results

Of the 616 patients entering the study between February 1982 and September 1985, six were subsequently excluded. These exclusions were due to wrong diagnosis (four) and previous malignancy within the preceding 5 years (two). Thus 610 patients were available for analysis. The characteristics of the 610 patients allocated at entry to each treatment are shown in Table I. In the whole study the median age was 62 years; 68.4% were male and 31.6% female; 196 (32.1%) had limited disease and 414 (67.9%) extensive disease.

The response rate (complete and partial) to the initial chemotherapy is shown in Table II. These response rates are given after four courses of chemotherapy in both groups and after a further four cycles in the long course group. After four courses of chemotherapy the response rates in the short

**Table I** Patients characteristics at presentation according to assigned treatment

| | *Short* | | | *Long* | | |
|---|---|---|---|---|---|---|
| | *At presentation (total)* | *According to 2nd randomisation* | | *At presentation* | *According to 2nd randomisation* | |
| | | *Chemo.* | *Sympt.* | | *Chemo.* | *Sympt.* |
| *n* | 305 | 144[a] | 145[a] | 305 | 150[a] | 145[a] |
| Lim/ext (%) | 31/69 | 34/66 | 32/68 | 33/67 | 35/65 | 32/68 |
| PS 0 and 1 | 71% | 75% | 69% | 77% | 78% | 77% |
| 2–4 | 29% | 25% | 31% | 23% | 22% | 23% |
| Age (years) | | | | | | |
| Median | 62 | 61 | 62 | 63 | 62 | 63 |
| range | (31–74) | (34–74) | (31–74) | (34–74) | (34–74) | (34–74) |
| M/F(%) | 67/33 | 65/35 | 69/31 | 70/30 | 73/27 | 68/32 |

[a]The total of patients who received a second randomisation is 584. This excludes 26 patients who were not randomised at the time of relapse, from the analysis of survival after relapse (see **statistical methods**).

**Table II** Response rates to initial chemotherapy; the reponse is recorded as the best response achieved during treatment

| | *Short* | | | *Long* | | | | | |
|---|---|---|---|---|---|---|---|---|---|
| | *Response by 4 courses* | | | *Response by 4 courses* | | | *Response by 8 courses* | | |
| | *n* | % | | *n* | % | | *n* | % | |
| Complete response | 37 | 12.1 | 61% | 27 | 8.9 | 61% | 46 | 15.1 | 63% |
| Partial response | 149 | 48.9 | | 159 | 52.1 | | 146 | 47.9 | |
| Stable disease | 64 | 21.0 | | 68 | 22.3 | | 62 | 20.3 | |
| Progressive disease (including deaths | 46 | 15.1 | | 41 | 13.4 | | 41 | 13.4 | |
| Inevaluable | 9 | 2.9 | | 10 | 3.3 | | 10 | 3.3 | |
| Total | 305 | | | 305 | | | 305 | | |

and long course chemotherapy groups were similar, and comparable numbers of patients showed progression of disease during chemotherapy. After eight cycles the overall response had not significantly increased but there was a small increase in proportion of complete responders.

Eighty-eight (29%) patients did not complete the four allocated courses of chemotherapy in the short course group and 152 (50%) patients failed to complete long course treatment. The reasons for failure are summarised in Table III. The resulting distribution of numbers of courses actually received for each intended randomisation is shown in Table IV.

During the second randomisation, 54 patients allocated to receive relapse chemotherapy after short course treatment failed to receive it and 70 patients similarly failed to receive allocated relapse chemotherapy after long course chemotherapy. The reasons are summarised in Table V. Ninety subjects went on to relapse chemotherapy after short course treatment and 80 did so following long course chemotherapy. Responses to relapse chemotherapy were low, with a total of 22.3% of patients showing a complete or partial response. There was no statistical difference in the response rate in the two groups, although the response rate was slightly higher for those who had received short course chemotherapy ($P=0.37$; Table VI) before their relapse chemotherapy.

The overall survival for all patients, based on the initial randomisation, is shown in Figure 1. There was no significant difference between the two survival curves ($P=0.085$), but median survival in the patients randomised to long course chemotherapy was 39 weeks, compared with 32 weeks in those receiving short course treatment (false negative $P=0.007$).

The combined results (Figure 2) show that of the four treatment policies, four courses of chemotherapy alone gives inferior survival to the other three treatments, which are equivalent in outcome. Thus, if chemotherapy is given on relapse, there is no survival disadvantage when initial treatment is stopped after four cycles. In contrast, in those patients receiving eight cycles of initial treatment, chemotherapy on relapse does not significantly improve survival compared with symptomatic treatment.

The progression-free interval was significantly longer for patients allocated to receive long course chemotherapy

**Table III** Reasons for not completing assigned initial chemotherapy

| | *Short* (*n*) | *Long* (*n*) |
|---|---|---|
| | 305 | 305 |
| Withdrawals | 13 | 26 |
| Disease progression (including deaths) during chemotherapy | 66 | 114 |
| Medical complications | 1 | 5 |
| Errors | 6 | 5 |
| Died before course 1 given | 2 | – |
| Given radiotherapy instead of 1st course | – | 2 |
| | 88 (29%) | 152 (50%) |

**Table IV** The number (and %) of patients stopping initial chemotherapy at each point in the assigned chemotherapy programme

| *Short* (*n*=*305*) | | | *Long* (*n*=*305*) | | |
|---|---|---|---|---|---|
| | *n* | % | | *n* | % |
| 0[a] | 4 | 1.3 | 0[a] | 2 | 0.6 |
| 1 | 46 | 15.1 | 1 | 45 | 14.8 |
| 2 | 15 | 4.9 | 2 | 12 | 3.9 |
| 3 | 21 | 6.9 | 3 | 15 | 4.9 |
| 4 | 217 | 71.1 | 4 | 24 | 7.9 |
| 5[b] | 2 | 0.7 | 5 | 15 | 4.9 |
| | | | 6 | 18 | 5.9 |
| | | | 7 | 20 | 6.6 |
| | | | 8 | 153 | 50.2 |
| | | | 9[a] | 1 | 0.3 |

[a]Randomised but not treated.
[b]Two patients treated in error.

**Table V** Reasons for patients not receiving assigned relapse chemotherapy

| | *Short course* | *Long course* |
|---|---|---|
| Deaths during initial chemotherapy | 19 | 24 |
| Patient refusals | 11 | 16 |
| Medical contraindications | 8 | 13 |
| Emergency radiotherapy | 3 | 7 |
| Died in remission | 1 | 0 |
| Not yet relapsed | 4 | 3 |
| Reasons not known | 5 | 7 |
| Protocol violation | 3 | 0 |
| | 54/144(37.5%) | 70/150(47%) |

**Table VI** Response to relapse chemotherapy following short or long course initial chemotherapy (short *vs* long, $P=0.37$)

| | *Short* | | | *Long* | | |
|---|---|---|---|---|---|---|
| | *n* | % | | *n* | % | |
| Complete response | 5 | 5.6 | 25.6 | 1 | 1.1 | 18.7 |
| Partial response | 18 | 20.0 | | 14 | 17.6 | |
| Stable disease | 39 | 43.3 | | 37 | 46.3 | |
| Progressive disease | 26 | 28.9 | | 28 | 35.0 | |
| Inevaluable | 2 | 2.2 | | 0 | 0 | |
| | 90 | | | 80 | | |

**Figure 1** Overall survival for all patients based on the initial randomisation. (A) Short course chemotherapy ($n=305$, MS 32 weeks); (B) long course chemotherapy ($n=305$, MS 39 weeks) ($p=0.085$, false negative $P=0.007$).

**Figure 2** Overall survival for all patients according to the four treatment intentions. (A) Short initial treatment, symptomatic relapse treatment ($n=145$, MS=30 weeks); (B) short initial treatment, chemotherapy on relapse ($n=144$, MS=38 weeks); (C) long initial treatment, symptomatic relapse treatment ($n=145$, MS=38 weeks); (D) long initial treatment, chemotherapy on relapse ($n=150$, MS 42 weeks). A *vs.* B $P=0.04$; A *vs.* C $P=0.05$; B *vs.* D $P=0.84$ (false negative $P<0.0001$).

**Figure 3** The progression free interval, for patients with stable and responding disease, following initial chemotherapy. Median interval following short (A) 23 weeks, $n=250$; long (B) 31 weeks, $n=254$; $P<0.001$. (Vertical bars indicate patients in whom the exact date of progression was not known.)

(Figure 3). During the initial three months of the study, when all patients were receiving chemotherapy, the rates of relapse were similar but the rate of progression increased in the short course group once chemotherapy had ceased. The median progression-free intervals were 23 and 31 weeks for short and long course patients respectively ($P<0.001$).

Figure 4 shows the survival curves from relapse to death. For patients treated with short or long course chemotherapy, who were randomised to receive symptomatic treatment only at relapse, the curves are identical with median survivals of 11 and 12 weeks. In the patients randomised to further chemotherapy at relapse, survival from relapse was better than with symptomatic treatment. Median survival was 15 weeks for those allocated to long course chemotherapy, and 20 weeks for those allocated to receive short course treatment. Comparing the four curves in Figure 4, survival from relapse was longer for those patients receiving short course and allocated to relapse chemotherapy.

When the effects of the treatment policies are separated according to disease extent the results are as shown in Table VII. Survival was adversely affected in patients with extensive disease who were treated with four cycles of initial chemotherapy only. A similar trend was observed for limited stage patients but was not statistically significant. In the

**Figure 4** The survival curves for relapse to death comparing short symptomatic (A) ($n=106$, MS-11 weeks), long and symptomatic (B) ($n=112$, MS=12 weeks), long and relapse chemotherapy (C) ($n=114$, MS=15 weeks), and short and relapse chemotherapy (D) ($n=105$, MS=20 weeks). A *vs.* D $P<0.001$. B *vs.* C $P=0.160$.

patients with limited disease the proportion of 24-month survivors was lowest in those treated with four chemotherapy cycles alone (although this was less apparent at 36 months). If the study is analysed further to include only the responding population, the disadvantage of giving four courses of chemotherapy alone becomes more apparent. Figure 5 shows the survival curves for the responding population randomised to receive short and symptomatic treatment versus long and symptomatic. The survival curve for the long treatment arm is significantly better ($P=0.01$) than for the short and a difference is still apparent at two years. The other two randomisations (Figure 6) show no difference for the responders receiving short and relapse or long and relapse chemotherapy (false negative $P=0.002$).

Toxicity data included all deaths considered to be directly attributable to drug toxicity. There were 18 in the short course and 11 in the long course chemotherapy arms. However, the death rate in early cycles is not randomly distributed during the inter-cycle period and we have shown, and will report separately, that it is likely that chemotherapy induced toxicity contributes to early death in patients with extensive disease. The number of reported episodes of serious infection (WHO grade 2) was 45 during short course chemotherapy and 70 during long. Recorded episodes of total white cell count falling below 3,000 or a neutropenia of 1,000 before a course of chemotherapy was 55 during short course and 182 during the long course treatment. Courses were delayed a total of 71 and 217 times during short and long course treatment respectively. Activity, mood, pain, nausea and vomiting were assessed in detail using daily diary cards as part of a study on quality of life which will be reported separately. In summary, mild nausea and vomiting occurred in almost all patients but continued longer in those receiving eight cycles of treatment. Hair loss was universal. Severe (WHO grade 2) mucositis occurred in 16 and 35 patients respectively, and neuropathy on four and 15 occasions during the short and long courses.

**Table VII** Median and 2-year survival related to disease extent presentation

| | *Median survival (weeks)* | *% 2-year survival* |
|---|---|---|
| *Limited disease* | | |
| Short | 43 | 9.5 |
| Long | 48 | 8.1 |
| Short + relapse CT | 44 | 14.7 |
| Short + symptomatic | 42 | 4.3 |
| Long + relapse CT | 44 | 7.9 |
| Long + symptomatic | 50 | 8.7 |
| *Extensive disease* | | |
| Short | 28 | 1.4 |
| Long | 35 | 1.7 |
| Short + relapse CT | 34 | 1.1 |
| Short + symptomatic | 28 | 2.0 |
| Long + relapse | 40 | 1.0 |
| Long + symptomatic | 34 | 2.7 |

In limited disease patients: short and symptomatic *vs* short and relapse CT $P=0.200$; *vs* long and symptomatic $P=0.10$; *vs* long and relapse CT $P=0.726$. In extensive disease patients: short and symptomatic *vs* short and relapse CT $P=0.11$; *vs* long and symptomatic $P=0.224$; *vs* long and relapse CT $P=0.01$.

**Figure 5** Overall survival of responding population receiving symptomatic treatment only at relapse. (A = short and symptomatic ($n=89$, MS = 40 weeks); B = long and symptomatic ($n=91$, MS = 49 weeks); $P=0.01$).

**Figure 6** Overall survival of responding population receiving relapse chemotherapy. (A = short and relapse ($n=95$, MS = 48 weeks); B = long and relapse ($n=97$, MS = 51 weeks); $P=0.42$).

### Discussion

This is one of the largest studies to address the effects of length of chemotherapy in patients with SCLC. The major referring centres within the multicentre group enter all patients presenting with the disease who are judged likely to survive a minimum of three weeks if left untreated, and the patients are therefore representative of the disease pattern within the community. Since the overall prognosis of SCLC is poor (Davis *et al.*, 1985) and many patients are elderly and of poor performance status, the duration of chemotherapy is an important practical consideration. There have been few studies addressing this question. Cullen *et al.* (1986) evaluated maintenance chemotherapy versus no treatment in patients with no unequivocal residual disease after induction therapy and found a 16-week advantage for continuing chemotherapy. In the EORTC study (Splinter *et al.*, 1986) five initial courses of chemotherapy were followed by a randomisation in the responding population either to a further seven courses of drug treatment or symptomatic treatment. There was no survival advantage for further chemotherapy in limited disease patients but a small advantage for extensive disease patients. There has been no study evaluating different durations of initial chemotherapy and the effect of further chemotherapy after relapse. The UK MRC Lung Cancer Working Party have compared six and 12 courses of chemotherapy and found no difference in median or 3-year survival (D. Girling, personal communication).

In our study, where treatment was stopped after only 12 weeks of chemotherapy in half the patients, the issue of what to do at relapse was important because the early cessation of therapy, especially in patients responding to treatment, may result in an unacceptably worse survival. For this reason we adopted a second randomisation which posed the question of the possible benefits of second line chemotherapy. The initial chemotherapy used widely accepted drugs active in small cell lung cancer (Hansen & Rørth, 1979). The second line drugs, methotrexate and doxorubicin, were of different classes, modes of action and are also active against the disease.

The results of this study show that continuing initial chemotherapy for eight courses does not greatly increase the response rate compared with four courses. However, stopping chemotherapy after four cycles resulted in earlier disease progression. At progression, there was a clear survival advantage for second line chemotherapy in those previously receiving short course chemotherapy, but this was less apparent in those given eight cycles. The overall study design therefore showed that four cycles of chemotherapy alone resulted in worse survival, but any of the other treatment policies equal. This disadvantage of short course chemotherapy alone was particularly obvious in the responding population both in median and 2-year survival.

The study analysis is based on intention to treat but it must be noted that many patients failed during initial chemotherapy and that the clinicians in charge sometimes felt that an individual was too ill to undergo relapse chemotherapy. Furthermore, some patients refused chemotherapy on relapse. Patients dropping out of the study are particularly likely to be patients with poor performance status and/or extensive disease.

The study has shown that intention to treat with chemotherapy at relapse was harder to realise with long course chemotherapy. Less than 50% of patients allocated to relapse chemotherapy following long course treatment were either willing or able to receive it. It can be concluded from this study that the policy of stopping treatment early will lead to earlier relapse. In the group as a whole no survival disadvantage results provided chemotherapy is given on relapse, and clinicians might judge that in some circumstances this may be a convenient treatment policy; for example in treating elderly and poor prognosis patients or in those with particularly severe side-effects of treatment. However, taken together with the other studies (Cullen *et al.*, 1986; Evans *et al.*, 1985; Splinter *et al.*, 1986) the present results show that there is a limit to the degree to which chemotherapy can be reduced without a deterioration in survival especially in those responding to treatment. The data from this study and from the MRC trial indicate that, for the majority of patients, six cycles of chemotherapy represents adequate treatment with combination chemotherapy programmes similar to those used in these two studies. Further improvements await new drug combinations and schedules for good prognosis limited disease patients and better palliative regimens for the others.

This study was supported by the Cancer Research Campaign. Walter Gregory gave invaluable help in statistical analysis. The computing facilities were made available by the Imperial Cancer Research Fund. Miss Angela Betchley and Miss Piera Cassettari typed the manuscript with great care. We would like to thank the following physicians for referring cases to this trial: Dr J.R. Govan, Dr G.H. Wiggins, Dr D. Phillips, Dr A. Willis, Dr C. Trask, Dr R. Weatherstone, Dr D.W. Empey, Dr G.W. Bradley, Dr J. Millard, Dr P. Cole, Dr J.V. Collins, Dr R.K. Knight, Dr M. Apps, Dr R.A. Storring, Dr J. Utting, Dr J. Warren, Dr P.R. Studdy, Dr J. Riordan, Dr M.W. McNicol, Dr J. Meadway, Dr M. Harrier, Dr W.A.C. McAllister, Dr M.E. Hodson, Dr A. Newman-Taylor, Dr R.A. Banks, Dr L.R. Bagg, Dr W.P.U. Kennedy, Dr I. Williams, Dr O. McCarthy, Dr N. Eiser, Dr G.M. Cochrane, Dr S. Steel, Dr M.R. Hetzel, Dr J. Milledge, Dr J. Waller, Dr M.E. Turner-Warwick, Dr M. Green and Dr A.H. Diamond.

### References

CULLEN, M. MORGAN, D., GREGORY, W. & 11 others (1986). Maintenance chemotherapy for anaplastic small cell carcinoma of the bronchus: a randomised controlled trial. *Cancer Chem. Pharmacol.*, **17**, 157.

DAVIS, S., WRIGHT, P.W., SCHULMAN, S.F., SCHOLES, D., THORNING, D. & HAMMAR S. (1985). Long-term survival in small-cell carcinoma of the lung: a population experience. *J. Clin. Oncol.*, **3**, 80.

EVANS, W.K., OSOBA, D., FELD, R., SHEPHERD, F.A., BAZOS, M.J. & DeBOER, G. (1985). Etoposide (VP-16) and cisplatin: an effective treatment for relapse in small cell lung cancer. *J. Clin. Oncol.*, **3**, 65.

FREEDMAN, L.S. (1982). Tables of the number of patients required in clinical trials using the Logrank test. *Stat. Med.*, **1**, 121.

HANSEN, H.H. & RØRTH, M. (1979). Lung cancer. In *Cancer Chemotherapy*, Pinedo, H.M. (ed.) p. 267. Excerpta Medica: Amsterdam.

HUGHES, D.J. (1981). Approximate error probabilities for the log rank test. Working paper of Research Centre for Mathematical Modelling of Clinical Trials, University of Warwick.

KAPLAN, E.L. & MEIER, P. (1958). Non parametric estimation from incomplete observations. *J. Am. Stat. Assoc.*, **53**, 457.

OSTERLIND, K. & ANDERSEN, P.K. (1986). A model for survival in small cell lung cancer. A study of prognostic factors in 874 patients treated with chemotherapy with or without irradiation. *Cancer Res.*, **46**, 4189.

PETO, R., PIKE, M.C., ARMITAGE, P. & 7 others (1977). Design and analysis of randomised clinical trials requiring prolonged observation of each patient. *Br. J. Cancer*, **35**, 1.

SOUHAMI, R.L., BRADBURY, I., GEDDES, D.M., SPIRO, S.G., HARPER, P.G. & TOBIAS, J.S. (1985). The prognostic significance of laboratory parameters measured at diagnosis in small cell carcinoma of the lung. *Cancer Res.*, **45**, 2878.

SOUHAMI, R.L., GEDDES, D.M., SPIRO, S.G. & 5 others (1984). Radiotherapy in small cell cancer of the lung treated with combination chemotherapy: a controlled trial. *Br. Med. J.*, **288**, 1643.

SPLINTER, T., McVIE, J.G., DELASSIO, O. & 20 others (1986). Induction versus induction plus maintenance chemotherapy in small cell lung cancer. *Am. Soc. Clin. Oncol.*, C188.